# Long-term multicomponent exercise enhances functional mobility in early-stage Parkinson’s disease: a 48-month retrospective cohort study

**DOI:** 10.3389/fphys.2025.1700510

**Published:** 2025-11-24

**Authors:** Marcos Moura, Marina Lívia Venturini Ferreira, Filipe Duo Speri, Lucas Celsius Matos Alves, João Vitor Cerávolo Rostirola, Edevaldo Donizetti de Campos, Miguel S. Conceição

**Affiliations:** 1 Health Science Program, Sao Francisco University, Bragança Paulista, Brazil; 2 Virtual University of the State of São Paulo, São Paulo, Brazil

**Keywords:** Parkinson’s disease, exercise therapy, functional mobility, TC6, TUG

## Abstract

**Background:**

Long-term effects of structured exercise on functional capacity in Parkinson’s disease (PD) remain poorly understood.

**Objective:**

To investigate the impact of a 48-month multicomponent exercise program on mobility, aerobic capacity, and muscle mass in individuals with early-stage PD, and to examine baseline predictors of functional performance.

**Methods:**

Thirty-three patients with PD (Hoehn & Yahr stages 1–2) participated in a supervised, thrice-weekly program including aerobic, resistance, balance, and flexibility exercises. Assessments at baseline and 48 months included the Timed Up and Go (TUG), Six-Minute Walk Test (6MWT), peak oxygen uptake (VO_2_peak), and muscle mass. Minimal detectable change (MDC), effect sizes, and linear regressions were computed.

**Results:**

After 48 months, TUG time decreased by −3.8 s (95% CI: −5.0 to −2.6; *p* < 0.001; *dz* = −1.12) and 6MWT distance increased by +88.8 m (95% CI: 45.1 to 133.0; *p* < 0.001; *dz* = 0.72), both exceeding MDC thresholds. VO_2_peak (−1.1 mL·kg^−1^·min^−1^; *p* = 0.142) and muscle mass (−0.7 kg; *p* = 0.068) showed no significant changes. VO_2_peak was the sole independent predictor of functional performance at baseline and follow-up, while muscle mass had limited explanatory value. Improvements were observed in both disease stages, with similar effect sizes.

**Conclusion:**

Long-term engagement in a multicomponent exercise program produces *sustained functional improvements exceeding measurement error* in mobility among individuals with early-stage PD, independent of changes in aerobic capacity or muscle mass. VO_2_peak consistently predicted functional outcomes, highlighting aerobic fitness as a key therapeutic target. These findings support the systematic integration of structured, multicomponent exercise into standard care protocols to preserve mobility and independence in PD.

## Highlights

- Sustained functional mobility gains: A 48-month multicomponent exercise program significantly improved mobility in early-stage Parkinson’s disease, with TUG and 6MWT changes exceeding minimal detectable change thresholds.
- VO_2_peak as a consistent predictor: Peak oxygen uptake remained the sole independent predictor of functional performance at baseline and follow-up, despite no significant absolute improvement, while muscle mass showed limited explanatory value.
- Clinical integration of exercise programs: Long-term, structured, multicomponent exercise should be incorporated into standard care protocols for early-stage Parkinson’s disease to help preserve mobility and independence.

## Introduction

Parkinson’s disease (PD) is the second most prevalent neurodegenerative disorder worldwide, and its incidence is rising with population aging (Ben-Shlomo et al., 2024). An estimated 8.5 million people live with PD globally, a number projected to double within the next-generation (Raket et al., 2022). In Brazil, where PD is not a notifiable condition (meaning that cases are not systematically reported to public health authorities), approximately 220,000 individuals are estimated to be affected (Bovolenta and Felicio, 2017). Given its high prevalence and progressive nature, understanding the underlying pathophysiology of PD is essential to guide therapeutic strategies.

PD involves progressive loss of dopaminergic neurons in the substantia nigra and striatal dopamine depletion, disrupting cortico-striatal networks (Poewe et al., 2017). Clinically, PD manifests with bradykinesia, rigidity, resting tremor, and postural/gait disturbances; non-motor symptoms include depression, autonomic dysfunction, psychosis, and cognitive decline (Simon et al., 2020; Tolosa et al., 2021). These motor and non-motor manifestations markedly reduce functional capacity, impair activities of daily living, and diminish overall quality of life (Arfa-Fatollahkhani et al., 2020; Carvalho et al., 2015). In addition, aging combined with neurodegeneration is associated with measurable physiological declines, including persistent weight loss, reduced muscle mass, and lower aerobic capacity, which further contribute to mobility limitations and functional deterioration (Pisciotta et al., 2020). These changes, alongside declines in aerobic capacity, can exacerbate mobility limitations and functional deterioration in PD. Reductions in physiological parameters such as maximal oxygen consumption (VO_2_peak), a key marker of aerobic fitness, and muscle mass are common in individuals with PD, have been proposed as markers of motor decline and physical deterioration ((Cai et al., 2021; Kanegusuku et al., 2016; Pechstein et al., 2020) and may contribute to impaired mobility and functional independence (Murphy and Lynch, 2023).

Current PD management relies on pharmacotherapy, particularly levodopa/carbidopa, which alleviates motor, and some non-motor, symptoms, especially in early stages (Armstrong and Okun, 2020). Despite initial benefits, pharmacological treatment loses effectiveness over time, and disease progression continues to impair functional capacity (Connolly and Lang, 2014). On the other hand, exercise is a core non-pharmacological therapy for individuals with PD (Alberts and Rosenfeldt, 2020).

Several studies have provided some evidence that exercises such as endurance training, resistance training, balance training and flexibility training have considerably reduced the motor signs of PD, regardless of the stage of the disease (Martignon et al., 2021). Exercise may help preserve body composition in people with PD, which is clinically relevant given the tendency toward weight and lean-mass loss with disease progression (Amato et al., 2022). Nevertheless, disease progression can erode performance over time, underscoring the need for ongoing clinical monitoring and sustained, stage-appropriate exercise strategies (Armstrong and Okun, 2020). Critically, it remains uncertain whether current exercise strategies combined with pharmacotherapy sustain functional capacity over multiple years.

Clinical performance tests create a map for detecting the domains affected by PD and can help to assess the different components of physical condition over time. The Timed Up and Go (TUG) test, encompassing sit-to-stand, walking, and turning, is a reliable indicator of functional mobility, correlating with fall risk, fear of falling, and functional performance in people with PD and healthy controls (Kluger et al., 2014; Morris et al., 2001). In parallel, the Six-Minute Walk Test (6MWT) evaluates aerobic capacity and gait performance in PD (Falvo and Earhart, 2009; Kobayashi et al., 2017). Understanding how functional performance evolves over extended periods is key to evaluating whether existing exercise strategies effectively support mobility and autonomy in individuals with PD. While VO_2_peak and muscle mass have been linked to motor decline, their roles as long-term predictors of functional outcomes remain underexplored. Clarifying this relationship may reveal mechanisms through which exercise promotes independence and help guide more precise interventions to preserve functional ability in this growing population.

Therefore, we hypothesized that long-term structured exercise would significantly improve functional performance in early-stage PD, and that baseline VO_2_peak and muscle mass would independently predict long-term functional capacity. This study investigated the effects of a four-year structured multicomponent exercise program on TUG and 6MWT performance in individuals with PD stages 1 and 2, and examined whether baseline VO_2_peak and muscle mass predicted individual variations in long-term functional performance. These findings may inform the development of targeted exercise interventions to preserve mobility and independence in people with PD.

## Methods

### Participants

Participants were identified from the center’s clinical database and invited to participate during routine visits. Eligible individuals were screened by a neurologist and an exercise physiologist to confirm the diagnosis and disease stage before inclusion. A total of 33 participants were recruited from the Raimunda Moura Program for parkinsonian (Atibaia, São Paulo, Brazil) between January and March 2018, and all follow-up assessments were completed by December 2022. The inclusion criteria for parkinsonian group were medical diagnosis of idiopathic PD, with a classification in 1 and 2 stage according to the modified Hoehn-Yahr scale (Opara et al., 2017). Exclusion criteria were the presence of unstable or decompensated cardiovascular conditions (e.g., recent myocardial infarction, uncontrolled arrhythmia) or severe musculoskeletal disorders that could preclude safe exercise testing or training were excluded. Exercise sessions and assessments took place continuously during this period at the Raimunda Moura Center Program for parkinsonian (Atibaia, São Paulo, Brazil). Attendance was recorded throughout the 48-month intervention, and participants were classified as consistent exercisers if they attended at least 50% of sessions annually. This adherence threshold was used to define the analytical cohort (n = 33).

This study was approved by the Ethical Research Committee of São Francisco University (number 5.791.053). Written informed consent was obtained from each participant. All patients went to multidisciplinary treatment in accordance with the program protocol and the project do not interfere in the clinical management.

### Study design

This investigation was embedded within an ongoing, community-based exercise program for individuals with Parkinson’s disease conducted at the Raimunda Moura Center (Atibaia, São Paulo, Brazil). The program follows an open-enrollment format, allowing participants to join or leave at any time. As part of routine clinical follow-up, comprehensive assessments were performed annually (baseline, 12, 24, 36, and 48 months), including evaluations of body composition, VO_2_peak, and functional performance (TUG and 6MWT).

The final sample included all participants who met inclusion criteria and completed 48 months of follow-up with full datasets. This analytical window (2018–2022) was selected retrospectively from the 10-year operation of the community-based exercise program, corresponding to the period with the largest number of participants who completed all annual evaluations. A formal power analysis was not performed, as the study represents a retrospective cohort derived from a pre-existing program. Therefore, the sample size reflects the available complete cases rather than an *a priori* estimation.

Only participants who completed four consecutive years of supervised exercise and all corresponding annual assessments were included in the analysis, ensuring complete longitudinal data across the 48-month period (n = 33). Because the analytical cohort was defined based on data completeness within an ongoing program, a conventional attrition rate was not applicable. Although annual evaluations were routinely performed, the statistical analysis focused on baseline and 48-month data to assess the cumulative long-term adaptations resulting from sustained participation in the exercise program, rather than transient changes between intermediate time points. This approach provides a clear estimate of the overall magnitude and clinical relevance of long-term exercise-induced changes in functional capacity and physiological parameters.

Clinical and demographic data were collected during the initial assessment. All assessments were conducted in the “on” medication state, approximately two hours after the participants’ regular morning dose of dopaminergic medication (predominantly levodopa-based regimens), to ensure consistent and comparable testing conditions across all time points. Exercise adherence was monitored through attendance records, and participants were classified as consistent exercisers if they attended at least 50% of the sessions over the 48 months, ensuring a minimum level of training exposure and data completeness for longitudinal analysis. Those who discontinued participation or had irregular attendance were excluded from the longitudinal analysis.

To minimize measurement bias, all assessments were conducted by the same trained evaluators using standardized protocols and calibrated equipment throughout the 48-month period. Although outcome assessors were not blinded to participant status, the use of objective measurement procedures and consistent testing conditions helped reduce the potential for systematic bias. This design enabled the evaluation of long-term functional adaptations to multicomponent exercise under real-world, community-based conditions. This study was conducted and reported in accordance with the Strengthening the Reporting of Observational Studies in Epidemiology (STROBE) guidelines for cohort studies (von Elm et al., 2007).

### Body composition

Body composition was assessed by a bioelectrical impedance analysis (BIA) instrument (InBody 230; BioSpace, Seoul, South Korea). The equipment uses a multifrequency system (10 impedance measurements at frequencies of 20 and 100 kHz) of tetrapolar electrodes with an eight-point tactile electrode. On the day of BIA measurements, volunteers were instructed to fast for 3 h (including water), empty their bladder beforehand (at least 30 min), abstain from strenuous exercise 24 h before the assessment and do not wear metallic accessories (i.e., watches, rings, earrings) during the test. After prior calibration, participants stood up on the equipment wearing light clothing to determine their body weight, in kilograms, and placed their hands and feet on the tactile electrodes of the instrument to measure impedance, according to the manufacturer’s instructions. Information on fat mass, skeletal muscle mass, fat-free mass and percentage of body fat was obtained using the Lookin’Body Software version LBM.1.2.0.16. Importantly, although DXA represents the gold standard for body composition assessment, BIA was used due to its accessibility and feasibility in the community-based setting. The method has shown good reliability and validity for estimating lean mass when standardized conditions are maintained.

### Maximum oxygen consumption test (VO_2peak_)

Maximum oxygen consumption was recorded during an incremental test carried out on an ergometric treadmill until exhaustion (Inbramed Super ATL). During the test, respiratory gas exchanges were collected and analyzed using the Fitmate-Pro (COSMED) equipment. For the test, participants were instructed to refrain from eating for 2 h, abstain from caffeine for 12 h, and normally follow the prescribed medication regimen. The test used the adapted Bruce protocol, consisting of 5 min of rest on the treadmill, followed by a 3-minute stage at a speed of 2.7 km/h without inclination and a subsequent 3-minute stage also at a speed of 2.7 km/h with a slope of 5%. The test was considered maximal when at least two of the following criteria were met: (1) respiratory exchange ratio (RER) ≥ 1.10; (2) heart rate ≥85% of age-predicted maximum; and (3) rating of perceived exertion ≥8 on the 0–10 Borg scale. This multi-criterion approach was adopted to account for potential autonomic dysfunction and blunted chronotropic response commonly observed in Parkinson’s disease. Accordingly, the highest value of oxygen uptake obtained during the test was considered VO_2_peak. All 33 participants (100%) completed the test to volitional.

### The six-minute walk distance test

A six-minute walk test was performed to assess aerobic endurance. Participants were instructed to walk as far as possible during a 6-minute period on a 30-m route. The route was identified by two cones, and the corridor was marked every 3 m according to ATS standards (Bohannon and Crouch, 2017). Likewise, the instructions and verbal encouragement given to the subjects were also standardized, being carried out every minute during the test until the subject was exhausted. The end of the test was determined by the participant’s voluntary exhaustion or the end of the pre-determined time.

### The timed up and go test

The Timed Up and Go (TUG) test was performed to assess the patients’ walking and transfer ability. Participants began the test sitting on a 46 cm high chair, with their arms extended over their thighs and their feet flat on the floor. At the evaluator’s signal, the individual should get up, without the help of their hands, and walk as quickly as possible, without running, around a cone positioned at a distance of three m from the chair, then returning to the starting position. The timer was started based on the evaluator’s signal and stopped at the moment the individual returned and sat down completely in the chair. Participants performed three attempts and their average was used for subsequent analysis (Bohannon and Crouch, 2017; Huang et al., 2011).

### Structured exercise program

Participants enrolled in the 48-month exercise program trained three times per week on alternate days, with sessions lasting approximately 60 min. The multicomponent training program included aerobic, resistance, balance, and flexibility exercises (Kim et al., 2019; Martignon et al., 2021). Sessions were performed in small groups of 6–10 participants under direct supervision of two exercise professionals in a community setting equipped with an outdoor walking track, resistance bands, and free weights. Each session began with six stretching exercises for large muscle groups (≈5 min), followed by 12 min of walking for the aerobic component. The intensity of the aerobic component was prescribed within 50%–80% of maximal heart rate (equivalent to 50%–70% of VO_2_peak), determined individually from each participant’s most recent VO_2_ test performed annually. Heart rate was continuously monitored using a heart rate monitor (Polar®, Kempele, Finland), and supervising professionals adjusted workload or pace to maintain participants within the target intensity zone. Thus, progression in aerobic training intensity was updated once per year according to the results of the annual cardiopulmonary exercise test. Subsequently, participants performed eight resistance exercises targeting upper and lower limbs, completing two sets of 20 repetitions with a one-minute rest between sets. Exercises were adapted to each participant’s motor stage, balance ability, and fatigue level, using individualized adjustments in range of motion and external load. Progression in resistance training was implemented pragmatically based on daily performance: when participants were able to complete all repetitions with apparent ease and correct form, the load or complexity of the movement was increased by the supervising professional. Fidelity to the prescribed multicomponent structure was ensured through continuous heart rate monitoring, direct supervision, and standardized session routines that consistently included all four components (aerobic, resistance, balance, and flexibility). Adjustments to aerobic training were made annually based on VO_2_peak reassessment, while modifications to resistance training occurred progressively during regular sessions, according to participant tolerance and performance. These adaptations maintained the fundamental structure and objectives of the program while reflecting its pragmatic, real-world nature. The design of the exercise program was guided by the American College of Sports Medicine (ACSM)(American College of Sports Medicine et al., 2009), which provides evidence-based recommendations for frequency, intensity, and type of training in older adults. All exercise sessions were supervised by qualified exercise professionals with formal training and experience in Parkinson’s disease rehabilitation. The intervention description was structured according to the Consensus on Exercise Reporting Template (Slade et al., 2016) to ensure transparent, replicable reporting of all components (frequency, intensity, type, equipment, setting, and supervision).

### Statistical analysis

Statistical analyses were performed using a quantitative approach. Normality was tested with the Shapiro–Wilk test, and outliers were detected using boxplots. For normally distributed variables, the paired Student’s t-test was applied; for non-normal distributions, the Wilcoxon signed-rank test was used. Changes from pre- to post-intervention were expressed as absolute mean differences (with 95% confidence intervals), relative percentage change, and effect sizes calculated as Cohen’s dz for paired comparisons (with 95% CI). A significance level of p < 0.05 was adopted. In addition, simple and multiple linear regression models were used to assess associations between VO_2peak_ and muscle mass (predictors) and functional performance (TUG and 6MWT as outcomes). Model fit was assessed using the coefficient of determination (R^2^) and p-values. Minimal detectable change (MDC) was also reported with 95% confidence. Analyses were conducted using Jamovi software (version 2.3.28). All variables included in the analysis contained complete data across the 48-month follow-up; thus, no missing values were present. Because this was a single-group longitudinal analysis with limited sample size and minimal between-subject variability in baseline characteristics (age, disease stage, and medication status), no covariates were included in the statistical models.

## Results

Baseline clinical characteristics are presented in Table 1. Thirty-three individuals with Parkinson’s disease completed the 48-month retrospective study (2018–2022), including 9 women (4 stage 1 and 5 stage 2) and 24 men (9 stage 1 and 15 stage 2).

**TABLE 1:** Patients with Parkinson’s disease clinical characteristics.

Characteristics of the subjects	Women (n = 9)	Men (n = 24)
Age (y)	64.44 (7.93)	69.75 (6.78)
Height (cm)	151.56 (5.66)	168.25 (6.72)
Weight (kg)	62.88 (14.94)	74.73 (14.38)
BMI (kg·m^-2^)	28.98 (7.50)	26.30 (4.67)
PD diagnosis duration (years) at baseline (2018)	1.67 (0.47)	2.38 (1.44)
PD diagnosis duration (years) after 48 months (2022)	5.67 (0.47)	6.38 (1.44)

BMI, Body mass index; PD, Parkinson disease. Data presented as mean (standard deviation).

After 48 months, TUG completion time decreased by −3.8 s (95% CI: −5.0 to −2.6; p < 0.001; dz = −1.12 [95% CI: −1.55 to −0.68]), and 6MWT distance increased by +88.8 m (95% CI: 45.1 to 133.0; p < 0.001; dz = 0.72 [95% CI: 0.33 to 1.10]) (Table 2). Muscle mass decreased by −0.7 kg (95% CI: −1.4 to 0.1; p = 0.068; dz = −0.33 [95% CI: −0.68 to 0.02]), and VO_2_peak changed by −1.1 mL·kg^−1^·min^−1^ (95% CI: −2.6 to 0.4; p = 0.142; dz = −0.26 [95% CI: −0.61 to 0.09]). Changes in TUG and 6MWT exceeded their respective MDC thresholds, whereas changes in VO_2_peak and muscle mass did not. Detailed results stratified by Hoehn & Yahr stage are provided in the Supplementary Material (Supplementary Tables S1, S2). At both baseline and post-intervention, TUG completion time was strongly and inversely correlated with 6MWT distance, both in the overall sample and within Hoehn & Yahr stages 1 and 2. Moderate associations with peak VO_2_ were also observed in some analyses, whereas no significant correlations were found for muscle mass. Detailed correlation coefficients for each time point and disease stage are available in the Supplementary Material (Supplementary Tables S3–S8).

**TABLE 2:** Functional and physiological performance of patients with Parkinson’s disease (Hoehn & Yahr stages 1 and 2; n = 33) in 2018 (pre) and 2022 (post) after 48 months of physical exercise intervention.

Outcomes	Pre	Post	Δ (%)	MD (IC95%)	P	Cohen’s dz (IC95%)	MDC
TUG	12.5 ± 4.4	8.7 ± 2.9	−30.14	−3.8 (−5.0 a −2.6)	<0.001^*^	−1.12 (−1.55, −0.68)	1.62
6MWT	387.3 ± 117.4	476.1 ± 134.3	22.93	88.8 (45.1 a 133.0)	<0.001^*^	0.72 (0.33, 1.10)	59.59
VO_2peak_	15.7 ± 4.0	14.6 ± 4.0	−7.12	−1.1 (−2.6 a 0.4)	0.142	−0.26 (−0.61, 0.09)	2.06
MM	27.3 ± 6.7	26.6 ± 6.1	−2.40	−0.7 (−1.4 a 0.1)	0.068	−0.33 (−0.68, 0.02)	0.96

Mean ± Standard Deviation; Δ (%) = percentage change; MD (95% CI) = absolute difference between time points with 95% confidence interval; Cohen’s d (95% CI) = effect size; MDC = minimal detectable change. *p < 0.05: statistically significant difference between pre- and post-intervention. TUG, Timed Up and Go test in seconds; 6MWT, Six-Minute Walk Test in meters; VO_2peak_, Peak oxygen uptake (mL × kg^−1^×min^−1^); MM, Muscle mass (Kg).

### Exploratory analyses

**FIGURE 1:**
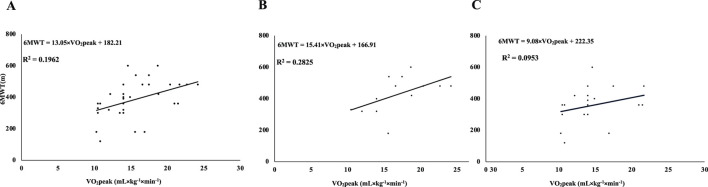
Linear regression between VO_2_peak and 6MWT distance in patients with Parkinson’s disease (Hoehn-Yahr stages 1 and 2) at baseline (2018). Simple linear regression models between estimated maximal oxygen uptake (VO_2_ peak) and the distance covered in the six-minute walk test (6MWT) at baseline (2018) in patients with Parkinson’s disease. Panel **(A)**: combined sample of patients at Hoehn-Yahr stages 1 and 2 (n = 33); Panel **(B)**: patients at Hoehn-Yahr stage 1 only (n = 13); Panel **(C)**: patients at Hoehn-Yahr stage 2 only (n = 20). Regression equations and corresponding coefficients of determination (R^2^) are presented.

Linear regression at baseline (2018), VO_2_peak explained 19.6% of the variance in 6MWT distance for the total sample (R^2^ = 0.196) (Figure 1A). Stratified analyses showed R^2^ = 0.283 for stage 1 (Figure 1B) and R^2^ = 0.095 for stage 2 (Figure 1C). Additional baseline regressions using muscle mass as predictor are presented in the Supplementary Figures (MM vs. 6MWT: Supplementary Figure S1; MM vs. TUG: Supplementary Figure S2).

**FIGURE 2:**
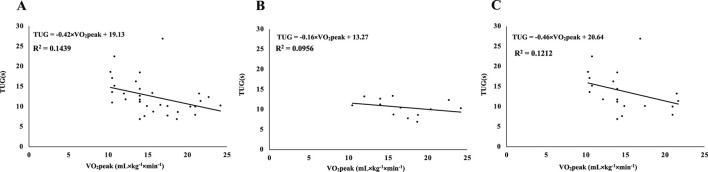
Linear regression between estimated peak oxygen uptake (VO_2_ peak) and Timed Up and Go (TUG) test performance in patients with Parkinson’s disease (Hoehn-Yahr stages 1 and 2) at baseline (2018). Simple linear regression models between estimated maximal oxygen uptake (VO_2_ peak) and performance time on the Timed Up and Go (TUG) test at baseline (2018) in patients with Parkinson’s disease. Panel **(A)**: combined sample of patients in Hoehn-Yahr stages 1 and 2 (n = 33); Panel **(B)**: patients in Hoehn-Yahr stage 1 only (n = 13); Panel **(C)**: patients in Hoehn-Yahr stage 2 only (n = 20). Regression line equations and coefficients of determination (R^2^) are presented.

For the association between VO_2_peak and TUG time, VO_2_peak explained 14.4% of the variance in the total sample (R^2^ = 0.144) (Figure 2A), with R^2^ = 0.096 for stage 1 (Figure 2B) and R^2^ = 0.121 for stage 2 (Figure 2C). Post-intervention regression models are shown in the Supplementary Figures (VO_2_peak vs. 6MWT: Supplementary Figure S3; VO_2_peak vs. TUG: Supplementary Figure S4; MM vs. 6MWT; Supplementary Figure S5; MM vs. TUG: Supplementary Figure S6).

The model of multiple linear regression baseline (2018) with 6MWT distance as the dependent variable (Table 3) indicated that VO_2_peak was the only significant predictor (β = 13.107, p = 0.011), whereas muscle mass was not significant (β = −0.347, p = 0.905). Complementary models are presented in the Supplementary Material: at baseline, VO_2_peak was also the only significant predictor of TUG performance (Supplementary Table S9; R^2^ = 0.148; β = −0.416, p = 0.035), with no contribution from muscle mass (p = 0.704); at post-intervention, VO_2_peak predicted both TUG time (Supplementary Table S10; R^2^ = 0.301; β = −0.372, p = 0.003) and 6MWT distance (Supplementary Table S11; R^2^ = 0.248; β = 14.590, p = 0.011), while muscle mass showed only a nonsignificant trend (p = 0.086 and p = 0.072, respectively).

**TABLE 3:** Multiple linear regression between VO_2_peak, muscle mass, and six-minute walk distance (6MWD) in patients with Parkinson’s disease (Hoehn-Yahr stages 1 and 2) at the pre-intervention time point (2018).

Predictor	Estimates	Standard error	T	P
Intercept	190.86	106.15	1.798	0.082
VO_2peak_	13.107	4.840	2.707	0.011*
MM	– 0.347	2.890	– 0.120	0.905

Multiple linear regression model with six-minute walk test (6MWT, in meters) distance as the dependent variable, and estimated maximal oxygen uptake (VO_2_peak, in mL × kg^−1^ × min^−1^) and muscle mass (MM, in kg) as independent variables at the pre-intervention time point (2018) in patients with Parkinson’s disease at Hoehn-Yahr stages 1 and 2 (n = 33). Regression coefficients (Estimates), standard errors, t-values, and p-values are presented. R^2^ represents the coefficient of determination of the model.

Regarding correlations across the full sample, TUG time was strongly and inversely correlated with 6MWT distance both at baseline (r = −0.78, p < 0.001) and after 48 months (r = −0.73, p < 0.001). Moderate associations were also observed between TUG and VO_2_peak (r = −0.38, p = 0.029 at baseline; r = −0.48, p = 0.005 post-intervention), and between 6MWT and VO_2_peak (r = 0.44, p = 0.010 at baseline; r = 0.40, p = 0.021 post-intervention). No significant correlations were found between muscle mass and any functional or aerobic variables. Full correlation are provided in Supplementary Tables S3–S8.

## Discussion

The present study demonstrates that consistent participation in a 48-month multicomponent exercise program led to significant long-term improvements in mobility among individuals with early-stage Parkinson’s disease. Both TUG and 6MWT performance improved beyond minimal detectable change thresholds, confirming sustained gains in functional capacity despite no significant increases in muscle mass or VO_2_peak. These findings suggest that enhanced neuromuscular coordination, rather than hypertrophic or aerobic adaptations, was the primary driver of improved mobility.

The observed magnitude and persistence of these functional gains extend previous evidence from shorter interventions (≤12 months) showing benefits of multicomponent or aerobic–resistance training in PD (Dibble et al., 2009; Frazzitta et al., 2015). Compared with those studies, the longer duration and pragmatic, community-based delivery of the present program likely contributed to sustained improvements, highlighting the long-term adherence and real-world applicability of supervised exercise in individuals with early-stage Parkinson’s disease.

These functional improvements may be explained, at least in part, by the participants’ early disease stages (Hoehn & Yahr 1 and 2), where the neural circuitry and peripheral systems retain a degree of plasticity that allows for meaningful adaptation in response to exercise (Kelly et al., 2018; Marino et al., 2023; Zikereya et al., 2023). At these stages, compensatory strategies and preserved motor pathways may still support gains in neuromotor coordination, gait efficiency, and postural control, all of which are critical for tasks like TUG and 6MWT (Giardini et al., 2018; Petzinger et al., 2013). Moreover, chronic engagement in multicomponent training, including aerobic, resistance, balance, and flexibility exercises, could have contributed to improved neuromuscular integration, greater movement confidence, and enhanced task-specific motor learning (Silva-Batista et al., 2016; Wong-Yu and Mak, 2015). It is plausible that these gains reflect both central and peripheral adaptations, such as increased cortical excitability or cerebellar compensation (Johansson et al., 2022; Li et al., 2022; Shah et al., 2016; Wang et al., 2025) and neuromuscular synchronization, characterized by improved timing and coordination of motor unit recruitment. Enhanced motor unit synchronicity and intermuscular coordination can lead to smoother, more efficient movement execution and reduced metabolic cost during gait or functional tasks, which may explain the improvements in TUG and 6MWT despite stable VO_2_peak (Burtscher et al., 2021; Jansen et al., 2021; Kelly et al., 2018; Zikereya et al., 2023). Similarly, the lack of significant muscle mass gain alongside improved functional performance supports the notion that long-term exercise primarily enhanced motor function and neuromuscular coordination rather than inducing hypertrophic adaptations. This interpretation is consistent with recent evidence demonstrating that resistance training–induced neuroplasticity can substantially contribute to motor performance improvements, often preceding or exceeding muscle hypertrophy (Hortobágyi et al., 2021). These authors highlight that neural adaptations—such as increased cortical excitability, motor unit recruitment efficiency, and intermuscular coordination, may play a central role in functional gains, even when morphological changes are modest. This reinforces the view that neural efficiency, rather than muscle quantity, was the main driver of mobility improvements observed after 4 years of training. Together, these findings suggest that sustained multicomponent training promotes functional and neuromuscular efficiency, allowing individuals with early-stage Parkinson’s disease to maintain mobility and independence even in the absence of measurable changes in maximal aerobic capacity.

It remains unclear whether similar benefits would be observed in patients with more advanced PD (stages 3 or 4), where postural instability, bradykinesia, and motor complications become more severe and may be less amenable to intervention. Studies in later-stage PD often show diminished responsiveness to interventions, highlighting the importance of early implementation of structured exercise programs (Yu et al., 2021). Thus, the improvements observed here underscore the therapeutic window during the early disease trajectory, when multicomponent training may yield the greatest returns in function and independence.

When stratified by disease severity, both Hoehn & Yahr stage 1 and stage 2 participants exhibited significant and sustained improvements in mobility after 48 months of structured multicomponent training. Improvements in TUG and 6MWT performance consistently exceeded minimal detectable change thresholds in both subgroups, indicating robust functional responsiveness to long-term exercise. Importantly, the magnitude of improvement was comparable between stages 1 and 2, suggesting that even individuals with mild-to-moderate motor impairment retain substantial plasticity and capacity for functional adaptation when engaged in sustained, supervised physical training. This suggests that disease progression, at least within early to moderate stages, does not necessarily preclude the efficacy of structured physical training. The improvements observed may stem from cumulative enhanced neuromotor learning, greater movement automaticity, and improved sensorimotor integration promoted by long-term exposure to task-specific training (Shen et al., 2016; Wang et al., 2025). Moreover, the lack of significant changes in VO_2_peak and muscle mass across both stages suggests that the functional improvements observed in our cohort were primarily mediated by neuromuscular adaptations, such as enhanced muscle strength (Shulman et al., 2013). While Shulman et al. (2013) (Shulman et al., 2013), reported improvements in VO_2_peak and 6MWT, the discrepancy may be explained by differences in intervention intensity, as their protocol involved a more vigorous exercise regimen. Collectively, these findings reinforce the role of exercise as a neurofunctional intervention, particularly in neurodegenerative conditions like Parkinson’s disease, where compensatory mechanisms may be essential for maintaining autonomy and Mobility.

Correlation analyses (Supplementary Tables S3–S8) revealed consistently strong inverse associations between TUG and 6MWT across all stages and time points (see Supplementary Tables S3–S8), indicating that better dynamic balance performance was tightly linked to greater walking capacity. These results highlight the importance of sustained engagement and adherence to physical training programs over time. Given that both tests capture aspects of real-world mobility and fall risk, their high interdependence highlights the robustness of these measures for tracking relevant functional change.

Notably, VO_2_peak was moderately and significantly associated with both functional outcomes, especially in the post-intervention phase (Supplementary Tables S6–S8). These associations reinforce the role of aerobic capacity in mobility and endurance but also highlight the contribution of other mechanisms, such as neuromotor adaptations or improved motor efficiency, to sustained functional improvements. This aligns with prior research indicating that aerobic capacity plays a supportive, though not exclusive, role in enhancing gait and endurance in PD (Shulman et al., 2013). In contrast, muscle mass was not significantly correlated with either TUG or 6MWT in any condition, which may reflect the fact that muscle quantity alone does not determine functional capacity in PD. Instead, neuromuscular efficiency, motor planning, and central drive may exert a stronger influence. These findings underscore the importance of targeting aerobic conditioning and neuromotor control over hypertrophy alone in exercise prescriptions for PD. While resistance training has documented benefits for strength (Shulman et al., 2013), its isolated effect on walking performance appears limited unless coupled with dynamic, functional, or task-specific exercises (Silva-Batista et al., 2016). This distinction is crucial when designing long-term interventions aimed at preserving independence and mobility in aging populations with neurodegenerative conditions.

Simple and multiple regression analyses offered further insights into the physiological determinants of functional capacity in PD. In the linear regressions, baseline VO_2peak_ was a moderate predictor of 6MWT performance and a weaker predictor of TUG, whereas muscle mass had minimal explanatory power. Following the intervention, the predictive strength of VO_2peak_ improved, particularly in patients at stage 2. These findings reinforce the notion that improvements in aerobic fitness, even in the absence of morphological changes, have functional significance. The persistently low explanatory power of muscle mass across models suggests that quantitative muscle preservation alone is insufficient to maintain mobility in PD, emphasizing the importance of targeting metabolic and neuromotor adaptations in exercise prescriptions.

Multiple regression models (Table 3; Supplementary Tables S9, S10) confirmed the independent contribution of VO_2peak_ to functional performance, reinforcing their role as a physiological marker. At both baseline and post-intervention, VO_2peak_ remained the only significant predictor of TUG and 6MWT outcomes, even after adjusting for muscle mass. Post-intervention models revealed significant associations between VO_2peak_ and both TUG and 6MWT, while muscle mass approached but did not reach significance. These results substantiate the hypothesis that aerobic conditioning plays a central role in functional capacity in PD. It is important to note that VO_2_peak values did not significantly increase over the 48 months; however, they consistently correlated with functional performance, reinforcing their role as a physiological marker.

This study has limitations, including the lack of a non-exercise control group, potential confounders related to medication or disease progression, the absence of muscle quality assessment, and the fact that exercise load and intensity were not systematically progressed. Although bioelectrical impedance analysis (BIA) is a practical and reliable tool for estimating body composition in community-based settings, it is important to acknowledge that this method may slightly overestimate fat-free mass and underestimate fat mass compared to DXA (Feng et al., 2024). However, as all measurements in the present study were performed under standardized conditions and using the same device and evaluators, the longitudinal comparisons remain valid and internally consistent. Although attendance was recorded, the limited sample size (n = 33) precluded additional exploratory analyses assessing whether the total number of attended sessions influenced the magnitude of functional improvements. Therefore, while all participants met the adherence threshold for consistent engagement, potential dose–response effects of training exposure could not be formally tested. Moreover, usual medical care and habitual physical activity outside the supervised program were not systematically recorded or controlled, which limits the ability to determine whether observed improvements were exclusively attributable to the exercise intervention. These limitations likely resulted in a conservative estimation of the true effects; for example, the inclusion of participants with variable adherence and medication regimens may have diluted potential exercise-related benefits, rather than exaggerating them. However, because the analytical cohort included only participants who maintained ≥50% attendance across 48 months, the findings may also reflect a selective subgroup with greater motivation, health stability, and access to consistent supervision. This selectivity could limit generalizability, as individuals with lower adherence or more advanced disease may experience smaller or less sustained benefits. Despite these limitations, the study’s strengths include its long duration of follow-up, adherence to standardized testing protocols, the use of both functional and physiological markers, and consistency across multiple statistical approaches, which collectively reinforce the validity of the findings. Future studies employing neuroimaging or electromyographic analyses could help clarify whether improvements in motor planning or cortico-subcortical connectivity underlie the functional gains observed.

These findings highlight the value of long-term, structured, multicomponent exercise programs in preserving mobility in early-stage PD. The consistent role of VO_2_peak as a predictor of functional performance suggests that maintaining aerobic fitness may be particularly relevant for this population. Given the absence of significant changes in muscle mass and the maintenance of functional performance, the present findings suggest that aerobic fitness may play a key role in sustaining functional capacity in individuals with early-stage Parkinson’s disease.

### Implications for research

The present findings demonstrate that long-term adherence to a community-based, multicomponent exercise program was feasible among individuals with early-stage Parkinson’s disease. Over the 48-month follow-up, participants who maintained regular attendance showed sustained improvements in mobility and functional capacity, even without significant changes in muscle mass or VO_2_peak. These results suggest that prolonged engagement in structured exercise may help maintain functional performance despite disease progression. Future research should build upon these findings by examining potential mediators of functional adaptation and by testing whether similar adherence and functional trajectories are observed in larger and more heterogeneous cohorts.

### Implications for clinical practice

This study indicates that long-term participation in a supervised, community-based multicomponent exercise program is achievable and may contribute to preserving mobility and independence in individuals with early-stage Parkinson’s disease. The consistency of functional improvements over 4 years underscores the value of maintaining regular exercise engagement early in the disease course. These findings highlight the practical relevance of sustained, structured exercise participation as a component of ongoing care for people with early-stage Parkinson’s disease.

## Conclusion

In conclusion, this 48-month longitudinal study suggests that consistent engagement in a multicomponent exercise program may sustained improvements in mobility among individuals with early-stage PD. VO_2_peak emerged as the key physiological correlate of functional capacity, supporting the potential importance of maintaining aerobic fitness to sustain functional independence. These findings contribute to the growing body of evidence advocating for the integration of long-term, structured, multicomponent exercise programs into the continuum of care for early-stage PD, while acknowledging that causal inference cannot be drawn from the present design. Because all participants were community-dwelling individuals with early-stage PD attending a single center, the generalizability of these results to patients with more advanced disease or limited access to supervised programs may be restricted.

## Data Availability

The raw data supporting the findings of this study will be made available by the authors upon request, without restriction.
