# Supplementary material for: Long-term multicomponent exercise enhances functional mobility in early-stage Parkinson’s disease: a 48-month retrospective cohort study
Source: Front Physiol. 2025 Nov 24;16:1700510. doi: 10.3389/fphys.2025.1700510 (PMC12682783; doi:10.3389/fphys.2025.1700510)
Supplement: Supplementary file 1 [file Table1.docx]

**Supplementary Material**

In participants at Hoehn & Yahr stage 1 (n = 13), TUG completion time decreased by 2.8 s (95% CI: –4.1 to –1.6; p < 0.001; dz = –1.38 [95% CI: –2.14 to –0.60]), exceeding the MDC threshold of 1.59 s, and 6MWT distance increased by 90.7 m (95% CI: 15.2 to 166.0; p = 0.022; dz = 0.73 [95% CI: 0.10 to 1.33]), not exceeding the MDC of 96.18 m. Muscle mass decreased by 0.5 kg (95% CI: –1.3 to 0.4; p = 0.254; dz = –0.33 [95% CI: –0.89 to 0.23]), and VO₂peak changed by –1.4 mL·kg⁻¹·min⁻¹ (95% CI: –4.2 to 1.4; p = 0.295; dz = –0.30 [95% CI: –0.85 to 0.26]), with neither exceeding their respective MDC thresholds (Table S1).

**Table S1.** Hoehn & Yahr Stage 1 participants (n = 13): pre- (2018) and post-intervention (2022) changes in functional performance and physiological variables.

|  | **Pre** | **Post** | **Δ (%)** | **MD (IC95%)** | **P** | **Cohen’s dz (IC95%)** | **MDC** |
| --- | --- | --- | --- | --- | --- | --- | --- |
| TUG | 10.5±2.1 | 7.7±1.8 | ̶ 27.11 | ̶ 2.8 (-4.1 a -1.6) | < 0.001^*^ | ̶ 1.38 (-2.14 a -0.60) | 1.59 |
| 6MWT | 428.5±115.7 | 519.2±128.1 | 21.18 | 90.7 (15.2 a 166.0) | 0.022 | 0.73 (0.10 a 1.33) | 96.18 |
| VO_2peak_ | 17.0±4.0 | 15.6±4.3 | ̶ 8.34 | ̶ 1.4 (-4.2 a 1.4) | 0.295 | ̶ 0.30 (-0.85 a 0.26) | 3.58 |
| MM | 25.4±5.7 | 24.9±6.1 | ̶ 1.82 | ̶ 0.5 (-1.3 a 0.4) | 0.254 | ̶ 0.33 (-0.89 a 0.23) | 1.07 |

Mean ± Standard Deviation; Δ (%) = percentage change; MD (95% CI) = absolute difference between time points with 95% confidence interval; Cohen’s d (95% CI) = effect size; MDC = minimal detectable change. p < 0.05: statistically significant difference between pre- and post-intervention. **TUG** = Timed Up and Go test; **6MWT** = Six-Minute Walk Test, **VO₂peak** = Peak oxygen uptake, **MM** = Muscle mass.

TUG decreased by 4.4 s (95% CI: –6.2 to –2.5; p < 0.001; dz = –1.11 [95% CI: –1.67 to –0.54]), exceeding the MDC of 2.43 s, and 6MWT increased by 87.5 m (95% CI: 28.8 to 146.0; p = 0.006; dz = 0.70 [95% CI: 0.20 to 1.18]), exceeding the MDC of 77.89 m. VO₂peak changed by –0.9 mL·kg⁻¹·min⁻¹ (95% CI: –2.8 to 1.0; p = 0.326; dz = –0.23 [95% CI: –0.67 to 0.22]) and muscle mass by –0.8 kg (95% CI: –1.9 to 0.3; p = 0.150; dz = –0.34 [95% CI: –0.78 to 0.12]), neither exceeding their MDC thresholds.

**Table S2.** Performance on TUG, 6MWT, peak VO₂, and muscle mass tests in patients with Parkinson’s disease (Hoehn & Yahr stage 2; n = 20) before (2018) and after (2022) 48 months of physical exercise intervention.

|  | **Pre** | **Post** | **Δ (%)** | **MD (IC95%)** | **P** | **Cohen’s dz (IC95%)** | **MDC** |
| --- | --- | --- | --- | --- | --- | --- | --- |
| TUG | 13.8±5.1 | 9.4±3.3 | ̶ 30.64 | ̶ 4.4 (-6.2 a -2.5) | < 0.001^*^ | ̶ 1.11 (-1.67 a -0.54) | 2.43 |
| 6MWT | 360.5±113.3 | 448.0±133.9 | 24.27 | 87.5 (28.8 a 146.0) | 0.006^*^ | 0.70 (0.20 a 1.18) | 77.89 |
| VO_2peak_ | 14.9±3.9 | 14.0±3.7 | ̶ 6.21 | ̶ 0.9 (-2.8 a 1.0) | 0.326 | ̶ 0.23 (-0.67 a 0.22) | 2.54 |
| MM | 28.6±7.1 | 27.8±6.3 | ̶ 2.73 | ̶ 0.8 (-1.9 a 0.3) | 0.150 | ̶ 0.34 (-0.78 a 0.12) | 1.44 |

Mean ± Standard Deviation; Δ (%) = percentage change; MD (95% CI) = absolute difference between time points with 95% confidence interval; Cohen’s d (95% CI) = effect size; MDC = minimal detectable change. p < 0.05: statistically significant difference between pre- and post-intervention. **TUG** = Timed Up and Go test; **6MWT** = Six-Minute Walk Test, **VO₂peak** = Peak oxygen uptake, **MM** = Muscle mass.

At Hoehn & Yahr stages 1 and 2 (n = 33), a strong negative correlation was observed between TUG and 6MWT (r = –0.780; p < 0.001), indicating that shorter TUG times were associated with greater walking distances. TUG also correlated inversely with peak VO₂ (r = –0.379; p = 0.029), and a positive correlation was found between 6MWT and peak VO₂ (r = 0.443; p = 0.010). No significant correlations were observed between muscle mass and the other variables.

**Table S3.** Correlation between performance on TUG, 6MWT, peak VO₂, and muscle mass at baseline (2018).

|  |  | TUG | 6MWT | VO_2peak_ | MM |
| --- | --- | --- | --- | --- | --- |
| TUG | Pearson’s r | ̶ |  |  |  |
|  | df | ̶ |  |  |  |
|  | p-value | ̶ |  |  |  |
| 6MWT | Pearson’s r | ̶ 0.780 | ̶ |  |  |
|  | df | 31 | ̶ |  |  |
|  | p-value | < 0.001^*^ | ̶ |  |  |
| VO_2pea_ | Pearson’s r | ̶ 0.379 | 0.443 | ̶ |  |
|  | df | 31 | 31 | ̶ |  |
|  | p-value | 0.029^*^ | 0.010 | ̶ |  |
| MM | Pearson’s r | ̶ 0.099 | 0.021 | 0.091 | ̶ |
|  | df | 31 | 31 | 31 | ̶ |
|  | p-value | 0.584 | 0.908 | 0.614 | ̶ |

Pearson correlation between functional tests—TUG (Timed Up and Go), 6MWT (Six-Minute Walk Test), estimated peak oxygen uptake (VO₂peak), and muscle mass (MM)—at the pre-intervention time point (2018) in patients with Parkinson’s disease at Hoehn & Yahr stages 1 and 2 (n = 33). Correlation coefficients (r), degrees of freedom (df), and p-values are presented. *p < 0.05: statistically significant value.

A strong negative correlation was observed between TUG and 6MWT (r = –0.818; p < 0.001), indicating that shorter TUG completion times were associated with greater walking distances. No other correlations reached statistical significance, including those between TUG and peak VO₂ (r = –0.309; p = 0.304), 6MWT and peak VO₂ (r = 0.531; p = 0.062), and all associations involving muscle mass **(Table S4).**

**Table S4.** Correlation between performance on TUG, 6MWT, peak VO₂, and muscle mass in patients with Parkinson’s disease (Hoehn & Yahr stage 1; n = 13) at baseline (2018**).**

|  |  | TUG | 6MWT | VO_2peak_ | MM |
| --- | --- | --- | --- | --- | --- |
| TUG | Pearson’s r | ̶ |  |  |  |
|  | df | ̶ |  |  |  |
|  | p-value | ̶ |  |  |  |
| 6MWT | Pearson’s r | ̶ 0.818 | ̶ |  |  |
|  | df | 11 | ̶ |  |  |
|  | p-value | < 0.001^*^ | ̶ |  |  |
| VO_2peak_ | Pearson’s r | ̶ 0.309 | 0.531 | ̶ |  |
|  | df | 11 | 11 | ̶ |  |
|  | p-value | 0.304 | 0.062 | ̶ |  |
| MM | Pearson’s r | 0.164 | 0.172 | 0.055 | ̶ |
|  | df | 11 | 11 | 11 | ̶ |
|  | p-value | 0.593 | 0.574 | 0.858 | ̶ |

Pearson correlation between functional tests—TUG (Timed Up and Go), 6MWT (Six-Minute Walk Test), estimated peak oxygen uptake (VO₂peak), and muscle mass (MM)—at the pre-intervention time point (2018) in patients with Parkinson’s disease at Hoehn & Yahr stage 1 (n = 13). Correlation coefficients (r), degrees of freedom (df), and p-values are presented.*p < 0.05: statistically significant value.

A strong negative correlation was observed between TUG and 6MWT (r = –0.810; p < 0.001), indicating that shorter TUG completion times were associated with greater walking distances. Correlations involving peak VO₂ (TUG: r = –0.348; p = 0.132; 6MWT: r = 0.309; p = 0.185) and muscle mass (TUG: r = –0.290; 6MWT: r = 0.056; peak VO₂: r = 0.222; all p > 0.05) were not statistically significant (Table S5).

**Table S5**. Correlation between performance on TUG, 6MWT, peak VO₂, and muscle mass in patients with Parkinson’s disease (Hoehn & Yahr stage 2; n = 20) at baseline (2018).

|  |  | TUG | 6MWT | VO_2peak_ | MM |
| --- | --- | --- | --- | --- | --- |
| TUG | Pearson’s r | ̶ |  |  |  |
|  | df | ̶ |  |  |  |
|  | p-value | ̶ |  |  |  |
| 6MWT | Pearson’s r | ̶ 0.810 | ̶ |  |  |
|  | df | 18 | ̶ |  |  |
|  | p-value | < 0.001^*^ | ̶ |  |  |
| VO_2peak_ | Pearson’s r | ̶ 0.348 | 0.309 | ̶ |  |
|  | df | 18 | 18 | ̶ |  |
|  | p-value | 0.132 | 0.185 | ̶ |  |
| MM | Pearson’s r | ̶ 0.290 | 0.056 | 0.222 | ̶ |
|  | df | 18 | 18 | 18 | ̶ |
|  | p-value | 0.214 | 0.815 | 0.347 | ̶ |

Pearson correlation between the functional tests TUG (Timed Up and Go), 6MWT (Six-Minute Walk Test), estimated peak oxygen consumption (VO₂peak), and muscle mass (MM) at the pre-intervention time point (2018) in patients with Parkinson’s disease at Hoehn & Yahr stage 2 (n = 20). Pearson’s correlation coefficients (r), degrees of freedom (df), and p-values are presented. *p < 0.05: statistically significant result.

TUG completion time was strongly and inversely associated with 6MWT distance (r = –0.733; p < 0.001) and moderately inversely associated with peak VO₂ (r = –0.477; p = 0.005). A moderate positive correlation was observed between 6MWT distance and peak VO₂ (r = 0.401; p = 0.021). No statistically significant correlations were found for muscle mass with any variable **(Table S6).**

**Table S6.** Correlation between performance on TUG, 6MWT, peak VO₂, and muscle mass in patients with Parkinson’s disease (Hoehn & Yahr stages 1 and 2; n = 33) at post-intervention (2022)**).**

|  |  | TUG | 6MWT | VO_2peak_ | MM |
| --- | --- | --- | --- | --- | --- |
| TUG | Pearson’s r | ̶ |  |  |  |
|  | df | ̶ |  |  |  |
|  | p-value | ̶ |  |  |  |
| 6MWT | Pearson’s r | ̶ 0.733 | ̶ |  |  |
|  | df | 31 | ̶ |  |  |
|  | p-value | < 0.001^*^ | ̶ |  |  |
| VO_2peak_ | Pearson’s r | ̶ 0.477 | 0.401 | ̶ |  |
|  | df | 31 | 31 | ̶ |  |
|  | p-value | 0.005^*^ | 0.021 | ̶ |  |
| MM | Pearson’s r | ̶ 0.222 | 0.253 | ̶ 0.100 | ̶ |
|  | df | 31 | 31 | 31 | ̶ |
|  | p-value | 0.215 | 0.155 | 0.579 | ̶ |

Pearson correlation between functional tests TUG (Timed Up and Go), 6MWT (Six-Minute Walk Test), estimated peak oxygen uptake (VO₂peak), and muscle mass (MM), at post-intervention (2022), in patients with Parkinson’s disease at Hoehn & Yahr stages 1 and 2 (n = 33). Presented are the correlation coefficients (r), degrees of freedom (df), and p-values. *p < 0.05: statistically significant result.

TUG completion time was strongly and inversely correlated with 6MWT distance (r = –0.860; p < 0.001), indicating that faster mobility was associated with greater walking capacity. No other correlations involving peak VO₂ or muscle mass were statistically significant **(Table S7).**

**Table S7.** Correlation between performance on TUG, 6MWT, peak VO₂, and muscle mass in patients with Parkinson’s disease (Hoehn & Yahr stage 1; n = 13) at post-intervention (2022**).**

|  |  | TUG | 6MWT | VO_2peak_ | MM |
| --- | --- | --- | --- | --- | --- |
| TUG | Pearson’s r | ̶ |  |  |  |
|  | df | ̶ |  |  |  |
|  | p-value | ̶ |  |  |  |
| 6MWT | Pearson’s r | ̶ 0.860 | ̶ |  |  |
|  | df | 11 | ̶ |  |  |
|  | p-value | < 0.001^*^ | ̶ |  |  |
| VO_2peak_ | Pearson’s r | ̶ 0.324 | 0.369 | ̶ |  |
|  | df | 11 | 11 | ̶ |  |
|  | p-value | 0.280 | 0.214 | ̶ |  |
| MM | Pearson’s r | ̶ 0.168 | 0.308 | ̶ 0.014 | ̶ |
|  | df | 11 | 11 | 11 | ̶ |
|  | p-value | 0.584 | 0.306 | 0.963 | ̶ |

Pearson correlation between functional tests TUG (Timed Up and Go), 6MWT (Six-Minute Walk Test), estimated maximal oxygen consumption (VO₂peak), and muscle mass (MM) at post-intervention (2022), in patients with Parkinson’s disease at Hoehn & Yahr stage 1 (n = 13). Correlation coefficients (r), degrees of freedom (df), and p-values are presented. *p < 0.05: statistically significant value.

TUG completion time was strongly and inversely correlated with 6MWT distance (r = –0.690; p < 0.001) and moderately with peak VO₂ (r = –0.530; p = 0.016). No other correlations, including those involving muscle mass, reached statistical significance (Table S8).

**Table S8.** Correlation between TUG, 6MWT, peak VO₂, and muscle mass in patients with Parkinson’s disease (Hoehn & Yahr stage 2; n = 20) at post-intervention (2022).

|  |  | TUG | 6MWT | VO_2peak_ | MM |
| --- | --- | --- | --- | --- | --- |
| TUG | Pearson’s r | ̶ |  |  |  |
|  | df | ̶ |  |  |  |
|  | p-value | ̶ |  |  |  |
| 6MWT | Pearson’s r | ̶ 0.690 | ̶ |  |  |
|  | df | 18 | ̶ |  |  |
|  | p-value | < 0.001^*^ | ̶ |  |  |
| VO_2peak_ | Pearson’s r | ̶ 0.530 | 0.371 | ̶ |  |
|  | df | 18 | 18 | ̶ |  |
|  | p-value | 0.016^*^ | 0.108 | ̶ |  |
| MM | Pearson’s r | ̶ 0.364 | 0.351 | ̶ 0.083 | ̶ |
|  | df | 18 | 18 | 18 | ̶ |
|  | p-value | 0.115 | 0.129 | 0.728 | ̶ |

Pearson correlation between functional tests TUG (Timed Up and Go), 6MWT (six-minute walk test), estimated maximal oxygen uptake (VO₂peak), and muscle mass (MM) at the post-intervention assessment (2022) in patients with Parkinson’s disease at Hoehn-Yahr stage 2 (n = 20). Correlation coefficients (r), degrees of freedom (df), and p-values are presented. *p < 0.05: statistically significant result.

Panel A: combined sample (Hoehn & Yahr stages 1 and 2; n = 33); Panel B: stage 1 only (n = 13); Panel C: stage 2 only (n = 20). At all levels, associations were not statistically significant, with models explaining <3% of variance (Panel A: R² = 0.0004; Panel B: R² = 0.0297; Panel C: R² = 0.0031) (Figure S1).

**Figure S1.** Simple linear regressions between muscle mass (MM) and six-minute walk test (6MWT) distance at baseline (2018) in patients with Parkinson’s disease.

**A B**

**6MWT = 0.37×MM + 377.29**

**6MWT(m)**

**R^2^ = 0.0297**

**6MWT = 3.47×MM + 340.46**

**MM(kg)**

**R^2^ = 0.0004**

**MM(kg)**

**6MWT(m)**

**C**

**6MWT = 0.90×MM + 334.90**

**R^2^ = 0.0031**

**6MWT(m)**

**MM(kg)**

**Simple linear regression models between muscle mass (MM) and six-minute walk test (6MWT) distance at baseline (2018) in patients with Parkinson’s disease.** Panel A: combined sample of patients in Hoehn-Yahr stages 1 and 2 (n = 33); Panel B: patients in Hoehn-Yahr stage 1 only (n = 13); Panel C: patients in Hoehn-Yahr stage 2 only (n = 20). Regression equations and coefficients of determination (R²) are presented.

Figure S2. Panel A: combined sample (Hoehn & Yahr stages 1 and 2; n = 33; R² = 0.0098); Panel B: stage 1 (n = 13; R² = 0.0268); Panel C: stage 2 (n = 20; R² = 0.0842). Associations were weak in all models, with no evidence of statistical or clinical relevance.

**Figure S2.** Simple linear regressions between muscle mass (MM) and Timed Up and Go (TUG) performance at baseline (2018) in patients with Parkinson’s disease.

**A B**

**MM(kg)**

**R^2^ = 0.0268**

**TUG = 0.06×MM + 9.01**

**TUG(s)**

**R^2^ = 0.0098**

**TUG = -0.07×MM + 14.29**

**MM(kg)**

**TUG(s)**

**C**

**TUG = -0.21×MM + 19.75**

**R^2^ = 0.0842**

**TUG(s)**

**MM(kg)**

**Simple linear regression models between muscle mass (MM) and Timed Up and Go (TUG) performance at baseline (2018) in patients with Parkinson’s disease.** Panel A: combined Hoehn-Yahr stages 1 and 2 (n = 33); Panel B: Hoehn-Yahr stage 1 only (n = 13); Panel C: Hoehn-Yahr stage 2 only (n = 20). Regression line equations and corresponding coefficients of determination (R²) are presented.

**Figure S3.** Panel A: combined sample (Hoehn & Yahr stages 1 and 2; n = 33; R² = 0.1609); Panel B: stage 1 (n = 13; R² = 0.1363); Panel C: stage 2 (n = 20; R² = 0.1373). All models indicated a moderate positive association between aerobic capacity and walking performance.

**Figure S3*.*** Simple linear regressions between estimated peak oxygen uptake (VO₂peak) and six-minute walk distance (6MWD) at post-intervention (2022) in patients with Parkinson’s disease.

**A B**

**R^2^ = 0.1609**

**R^2^ = 0.1363**

**6MWT(m)**

**VO_2_peak (mL×kg^-1^×min^-1^)**

**6MWT = 11.08×VO_2_peak + 346.81**

**6MWT(m)**

**6MWT = 13.58×VO_2_peak + 277.95**

**VO_2_peak (mL×kg^-1^×min^-1^)**

**C**

**6MWT = 13.28×VO_2_peak + 262.58**

**R^2^ = 0.1373**

**6MWT(m)**

**VO_2_peak (mL×kg^-1^×min^-1^)**

**Simple linear regression models between estimated peak oxygen uptake (VO₂peak) and six-minute walk distance (6MWD) at the post-intervention time point (2022) in patients with Parkinson’s disease.** Panel A: combined Hoehn-Yahr stages 1 and 2 (n = 33); Panel B: Hoehn-Yahr stage 1 only (n = 13); Panel C: Hoehn-Yahr stage 2 only (n = 20). Regression equations and corresponding coefficients of determination (R²) are presented.

**Figure S4.** Panel A: combined sample (Hoehn & Yahr stages 1 and 2; n = 33; R² = 0.2279); Panel B: stage 1 (n = 13; R² = 0.1050); Panel C: stage 2 (n = 20; R² = 0.2814). A moderate negative association was observed in the total sample, stronger in stage 2, while in stage 1 the association was weak and non-significant.

**Figure S4.** Simple linear regressions between estimated peak oxygen uptake (VO₂peak) and Timed Up and Go (TUG) performance at post-intervention (2022) in patients with Parkinson’s disease.

**A B**

**R^2^ = 0.1050**

**TUG = -0.14×VO_2_peak + 9.80**

**TUG(s)**

**VO_2_peak (mL×kg^-1^×min^-1^)**

**R^2^ = 0.2279**

**TUG(s)**

**TUG = -0.35×VO_2_peak + 13.86**

**VO_2_peak (mL×kg^-1^×min^-1^)**

**C**

**TUG = -0.47×VO_2_peak + 16.02**

**R^2^ = 0.2814**

**TUG(s)**

**VO_2_peak (mL×kg^-1^×min^-1^)**

**Simple linear regression models between estimated peak oxygen consumption (VO₂peak) and Timed Up and Go (TUG) test performance at the post-intervention time point (2022) in patients with Parkinson’s disease.** Panel A: combined sample of Hoehn-Yahr stages 1 and 2 (n = 33); Panel B: patients at Hoehn-Yahr stage 1 only (n = 13); Panel C: patients at Hoehn-Yahr stage 2 only (n = 20). Regression equations and respective coefficients of determination (R²) are presented.

Figure S5. Panel A: combined Hoehn & Yahr stages 1 and 2 (n = 33; R² = 0.0642); Panel B: stage 1 (n = 13; R² = 0.0949); Panel C: stage 2 (n = 20; R² = 0.1231). All associations were weak and non-significant.

**Figure S5.** Simple linear regressions between muscle mass (MM) and six-minute walk distance (6MWD) at post-intervention (2022) in patients with Parkinson’s disease.

**A B**

**6MWT = 7.25×MM + 338.77**

**6MWT(m)**

**R^2^ = 0.0949**

**MM(kg)**

**R^2^ = 0.0642**

**MM(kg)**

**6MWT = 5.59×MM + 326.98**

**6MWT(m)**

**C**

**6MWT = 7.41×MM + 242.29**

**R^2^ = 0.1231**

**MM(kg)**

**6MWT(m)**

**Simple linear regression models between muscle mass (MM) and six-minute walk distance (6MWD) at the post-intervention time point (2022) in patients with Parkinson’s disease.** Panel A: combined sample of patients at Hoehn-Yahr stages 1 and 2 (n = 33); Panel B: patients at Hoehn-Yahr stage 1 only (n = 13); Panel C: patients at Hoehn-Yahr stage 2 only (n = 20). Regression line equations and respective coefficients of determination (R²) are presented.

Figure S6. Panel A: combined Hoehn & Yahr stages 1 and 2 (n = 33; R² = 0.0492); Panel B: stage 1 (n = 13; R² = 0.0281); Panel C: stage 2 (n = 20; R² = 0.1326). All models showed weak, non-significant negative associations.

**Figure S6.** Simple linear regressions between muscle mass (MM) and Timed Up and Go (TUG) completion time at post-intervention (2022) in patients with Parkinson’s disease.

**A B**

**R^2^ = 0.0281**

**TUG = -0.06×MM + 9.05**

**TUG(s)**

**MM(kg)**

**R^2^ = 0.0492**

**MM(kg)**

**TUG(s)**

**TUG = -0.11×MM + 11.57**

**C**

**TUG = -0.19×MM + 14.72**

**R^2^ = 0.1326**

**TUG(s)**

**MM(kg)**

**Simple linear regression models between muscle mass (MM) and Timed Up and Go (TUG) completion time at the post-intervention time point (2022) in patients with Parkinson’s disease.** Panel A: combined sample of patients at Hoehn-Yahr stages 1 and 2 (n = 33); Panel B: patients at Hoehn-Yahr stage 1 only (n = 13); Panel C: patients at Hoehn-Yahr stage 2 only (n = 20). Regression line equations and respective coefficients of determination (R²) are presented.

**Table S9.** Multiple linear regression between estimated maximal oxygen uptake (VO₂peak) and muscle mass (MM) as predictors of Timed Up and Go (TUG) completion time (seconds) at the pre-intervention time point (2018) in patients with Parkinson’s disease (Hoehn–Yahr stages 1 and 2; n = 33). The model equation was:
TUG = –0.416 × VO₂peak – 0.043 × MM + 20.21 (R² = 0.148).

Only VO₂peak was a significant predictor (p = 0.035), indicating that higher aerobic capacity was associated with shorter TUG time. Muscle mass was not significant (p = 0.704).

**Table S9.** Multiple linear regression between VO₂peak, muscle mass, and Timed Up and Go (TUG) completion time in patients with Parkinson’s disease (Hoehn-Yahr stages 1 and 2) at the pre-intervention time point (2018).

| **Predictor** | **Estimates** | **Standard Error** | **t** | **p** |
| --- | --- | --- | --- | --- |
| Intercept | 20.21 | 4.133 | 4.889 | < 0.001^*^ |
| VO_2peak_ | ̶ 0.416 | 0.189 | ̶ 2.206 | 0.035^*^ |
| MM | ̶ 0.043 | 0.112 | ̶ 0.383 | 0.704 |

**Multiple linear regression model with Timed Up and Go (TUG, in seconds) completion time as the dependent variable, and estimated maximal oxygen uptake (VO₂peak, in kL×kg^-1^×min^-1^) and muscle mass (MM, in kg) as independent variables, at the pre-intervention time point (2018), in patients with Parkinson’s disease at Hoehn-Yahr stages 1 and 2 (n = 33).** Regression coefficients (Estimates), standard errors, t-values, and p-values are presented. R² represents the coefficient of determination of the model. *Statistically significant predictor (p < 0.05).

**Table S10.** Multiple linear regression between estimated maximal oxygen uptake (VO₂peak) and muscle mass (MM) as predictors of Timed Up and Go (TUG) completion time at the post-intervention time point (2022) in patients with Parkinson’s disease (Hoehn–Yahr stages 1 and 2; n = 33).
The model explained 30.1% of the variance (R² = 0.301), with the equation:
TUG = –0.372 × VO₂peak – 0.131 × MM + 17.64.
VO₂peak was a significant predictor (p = 0.003), while MM showed a trend toward significance (p = 0.086). The intercept (17.64) was significant (p < 0.001).

**Table S10.** Multiple linear regression between VO₂peak, muscle mass, and Timed Up and Go (TUG) completion time in patients with Parkinson’s disease (Hoehn-Yahr stages 1 and 2) at the post-intervention time point (2022).

| **Predictor** | **Estimates** | **Standard Error** | **T** | **p** |
| --- | --- | --- | --- | --- |
| Intercept | 17.64 | 2.723 | 6.480 | < 0.001* |
| VO_2peak_ | ̶ 0.372 | 0.113 | ̶ 3.290 | 0.003* |
| MM | ̶ 0.131 | 0.074 | ̶ 1.780 | 0.086 |

**Multiple linear regression model with Timed Up and Go (TUG, in seconds) completion time as the dependent variable, and estimated maximal oxygen uptake (VO₂_peak_, in** kL×kg^-1^×min^-1^**) and muscle mass (MM, in kg) as independent variables, at the post-intervention time point (2022), in patients with Parkinson’s disease at Hoehn-Yahr stages 1 and 2 (n = 33).** Regression coefficients (Estimates), standard errors, t-values, and p-values are presented. R² represents the coefficient of determination of the model. *Statistically significant predictor (p < 0.05).

**Table S11.** Multiple linear regression between estimated maximal oxygen uptake (VO₂peak) and muscle mass (MM) as predictors of six-minute walk test (6MWT) distance at the post-intervention time point (2022) in patients with Parkinson’s disease (Hoehn–Yahr stages 1 and 2; n = 33).
The model explained 24.8% of the variance (R² = 0.248), with the equation:
6MWT = 14.590 × VO₂peak + 6.550 × MM + 88.76.
VO₂peak was a significant predictor of walking distance (p = 0.011), while MM showed a nonsignificant trend (p = 0.072). The intercept (88.76) was not statistically significant (p = 0.499).

**Table S11.** Multiple linear regression between VO₂peak, muscle mass, and six-minute walk test (6MWT) distance in patients with Parkinson’s disease (Hoehn-Yahr stages 1 and 2) at the post-intervention time point (2022).

| **Predictor** | **Estimates** | **Standard Error** | **T** | **P** |
| --- | --- | --- | --- | --- |
| Intercept | 88.76 | 129.820 | 0.584 | 0.499 |
| VO_2peak_ | 14.590 | 5.390 | 2.707 | 0.011* |
| MM | 6.550 | 3.510 | 1.864 | 0.072 |

**Multiple linear regression model with six-minute walk test (6MWT, in meters) distance as the dependent variable, and estimated maximal oxygen uptake (VO₂peak, in kL×kg^-1^×min^-1^) and muscle mass (MM, in kg) as independent variables, at the post-intervention time point (2022), in patients with Parkinson’s disease at Hoehn-Yahr stages 1 and 2 (n = 33).** Regression coefficients (Estimates), standard errors, t-values, and p-values are presented. R² represents the coefficient of determination of the model. *Statistically significant predictor (p < 0.05).
